# The interference of Notch1 target Hes1 affects cell growth, differentiation and invasiveness of glioblastoma stem cells through modulation of multiple oncogenic targets

**DOI:** 10.18632/oncotarget.15013

**Published:** 2017-02-02

**Authors:** Carlo Cenciarelli, Hany E. Marei, Manuela Zonfrillo, Patrizia Casalbore, Armando Felsani, Stefano Giannetti, Gianluca Trevisi, Asma Althani, Annunziato Mangiola

**Affiliations:** ^1^ Institute of Translational Pharmacology (IFT), National Research Council (CNR), Roma, Italy; ^2^ Biomedical Research Center, Qatar University, Doha, Qatar; ^3^ Institute of Cell Biology and Neurobiology (IBCN), National Research Council (CNR), Roma, Italy; ^4^ Institute of Anatomy and Cell Biology, Catholic University-School of Medicine, Roma, Italy; ^5^ Department of Head and Neck, Institute of Neurosurgery, Catholic University-School of Medicine, Roma, Italy

**Keywords:** glioblastoma stem cells, Notch1, Hes1, self-renewal, differentiation

## Abstract

The invasive and lethal nature of Glioblastoma multiforme (GBM) necessitates the continuous identification of molecular targets and search of efficacious therapies to inhibit GBM growth. The GBM resistance to chemotherapy and radiation it is attributed to the existence of a rare fraction of cancer stem cells (CSC) that we have identified within the tumor core and in peritumor tissue of GBM. Since Notch1 pathway is a potential therapeutic target in brain cancer, earlier we highlighted that pharmacological inhibition of Notch1 signalling by γ-secretase inhibitor-X (GSI-X), reduced cell growth of some c-CSC than to their respective p-CSC, but produced negligible effects on cell cycle distribution, apoptosis and cell invasion. In the current study, we assessed the effects of Hes1-targeted shRNA, a Notch1 gene target, specifically on GBM CSC refractory to GSI-X. Depletion of Hes1 protein induces major changes in cell morphology, cell growth rate and in the invasive ability of shHes1-CSC in response to growth factor EGF. shHes1-CSC show a decrease of the stemness marker Nestin concurrently to a marked increase of neuronal marker MAP2 compared to pLKO.1-CSC. Those effects correlated with repression of EGFR protein and modulation of Stat3 phosphorylation at Y705 and S727 residues. In the last decade Stat3 has gained attention as therapeutic target in cancer but there is not yet any approved Stat3-based glioma therapy. Herein, we report that exposure to a Stat3/5 inhibitor, induced apoptosis either in shHes1-CSC or control cells. Taken together, Hes1 seems to be a favorable target but not sufficient itself to target GBM efficaciously, therefore a possible pharmacological intervention should provide for the use of anti-Stat3/5 drugs either alone or in combination regimen.

## INTRODUCTION

Among the different types of brain tumors, glioblastoma multiforme (GBM) is the most aggressive and recurrent one. GBM progression and recurrence often correlated with the existence of cancer stem cells (CSC), a small population of tumor initiating cells that have an enhanced resistance to radio- and chemotherapy [1].

Different molecular signaling pathways are involved in the uncontrolled GBM growth. Few years ago, the Cancer Genome Atlas (TCGA) proposed a gene expression-based classification of GBM: classical/EGFR+, proneural/PDGFR +, and mesenchymal/NF1+ [2, 3]. Such a classification has been crucial to explain the biology and the heterogeneous nature of GBM, but its prognostic value was rather limited. Recently, few reports suggest a mechanism-based classification that point to the identification of new prognostic markers not captured by existing classifications. Fifty percent of all GBM tumors present in the TCGA can be classified into high miR-21/low Sox2 or low miR-21/high Sox2 subtypes [4]. This classification can predict patient survival better than the currently used parameters. Recently, it has been reported that GBM progression and recurrence of a subset of tumors is associated with an increase of mesenchymal markers staining and aggressive behavior [5]. Notably, a bioinformatics analyses revealed a prominent role of Stat3 in GBM mesenchymal subtype and suggest an association between Notch1 and Stat3 signaling in this specific subtype [6]. Recently, it has been reported that MLK4 dependent-NF-κB activation induced a mesenchymal trans-differentiation and radio-resistance in GBM CSC, assigning to MLK4-NF-κB signaling axis a potential prognostic value useful for GBM with a mesenchymal signature [7, 8].

The Notch pathway, a therapeutic target in cancer, is known to play a crucial role in controlling cancer stem cell renewal, differentiation, apoptosis and angiogenesis [9–11]. The Notch pathway encompasses four types of receptors (Notch1, 2, 3, 4), and five membrane proteins ligands that include Delta-like ligands (DLL), 1, 2, 3, and Serrate/Jagged (JAG) 1 and 2. Binding of Notch ligands, and receptors located on the surfaces of neighbor cells, trigger the activation of cleavage enzymes such as ADAM10/TACE, and presenilin-dependent γ-secretase protease complex [12]. Following its cleavage, the Notch Intracellular Domain (NICD) is released into the nucleus, binds to transcription factor RBP-Jκ, and becomes an active transcriptional complex that induce the transcription of Notch target genes: Hairy Enhancer of Split 1 (HES1), HES-related proteins (HEY), p21 (CDKN1A), Cyclin D1(CCDN1), c-myc (MYC), BCL2, DTK1 (Deltex1) and NF-κB2 [13–16].

Beside Notch signaling, receptors tyrosine kinases (RTKs) such as epidermal growth factor receptor (EGFR) and platelet-derived growth factor receptor (PDGFR) are important contributors to GBM initiation and progression. RTKs activation through binding to specific ligands induces its auto-phosphorylation and activation of MAP kinase, PI3K/Akt, Jak/Stat3 signaling [17–20].

The concept of molecular therapy for GBM depends on possible genetic or pharmacological manipulations of the key signaling pathways with an ultimate goal of attenuating or inhibiting GBM growth and angiogenesis [9]. Evidence have demonstrated that Notch1 regulates transcription of EGFR through p53 [21]. Previous studies proved that targeting of PDGFRβ or PDGFRα attenuated self-renewal, survival, growth, epithelial to mesenchymal transition and angiogenesis of GBM CSC [22, 16].

In the current study, we aim to continue our previous efforts to elucidate the complex interplay between the major signaling pathways that boost GBM CSC growth. Specifically, we have investigated the effects of lentiviral transduction of Hes1-targeted shRNA in GBM peritumor-derived CSC resistant to GSI-X. We report a significant decrease of cell growth rate, changes in cell cycle distribution, attenuation of stem cells features and induction to neuronal differentiation. Depletion of Hes1 downregulates EGFR protein and modulates Stat3 phosphorylation at Tyr-705 and Ser-727. Herein, we identified Stat as a potential therapeutic target for GBM, indeed we report that the treatment with a Stat3/5 inhibitor, drives CSC to apoptosis. Taken together, our results provide a clear evidence that manipulation of Notch signaling pathways through inhibition of a downstream gene target, Hes1, is a favorable therapeutic strategy. Application of this strategy within the context of a more comprehensive multi-targeted pharmacological and/or genetic therapeutic regimen would expected to provide a more effective anti-GBM therapy.

## RESULTS

In this study, we investigated the effects of depletion of Hes1 expression, as one of the main target gene of Notch signaling, on the modulation of cell growth, differentiation, invasive ability and apoptosis of GBM CSC. GBM lesions were excised together with 1–2 cm from the tumor outer limit according to criteria previously provided [23]. Two types of CSC populations were derived from the excised GBM lesions: core-Cancer Stem Cells (c-CSC) and peritumor tissue-derived Cancer Stem Cells (p-CSC). Here, we concentrated specifically on a case of CSC (p-CSC1) that we previously demonstrated their marked resistance to apoptosis following their exposure to anti-Notch1/anti-EGFR drugs intervention [17].

### Hes1-directed shRNA affects major functional cellular pathways

**Figure 1:**
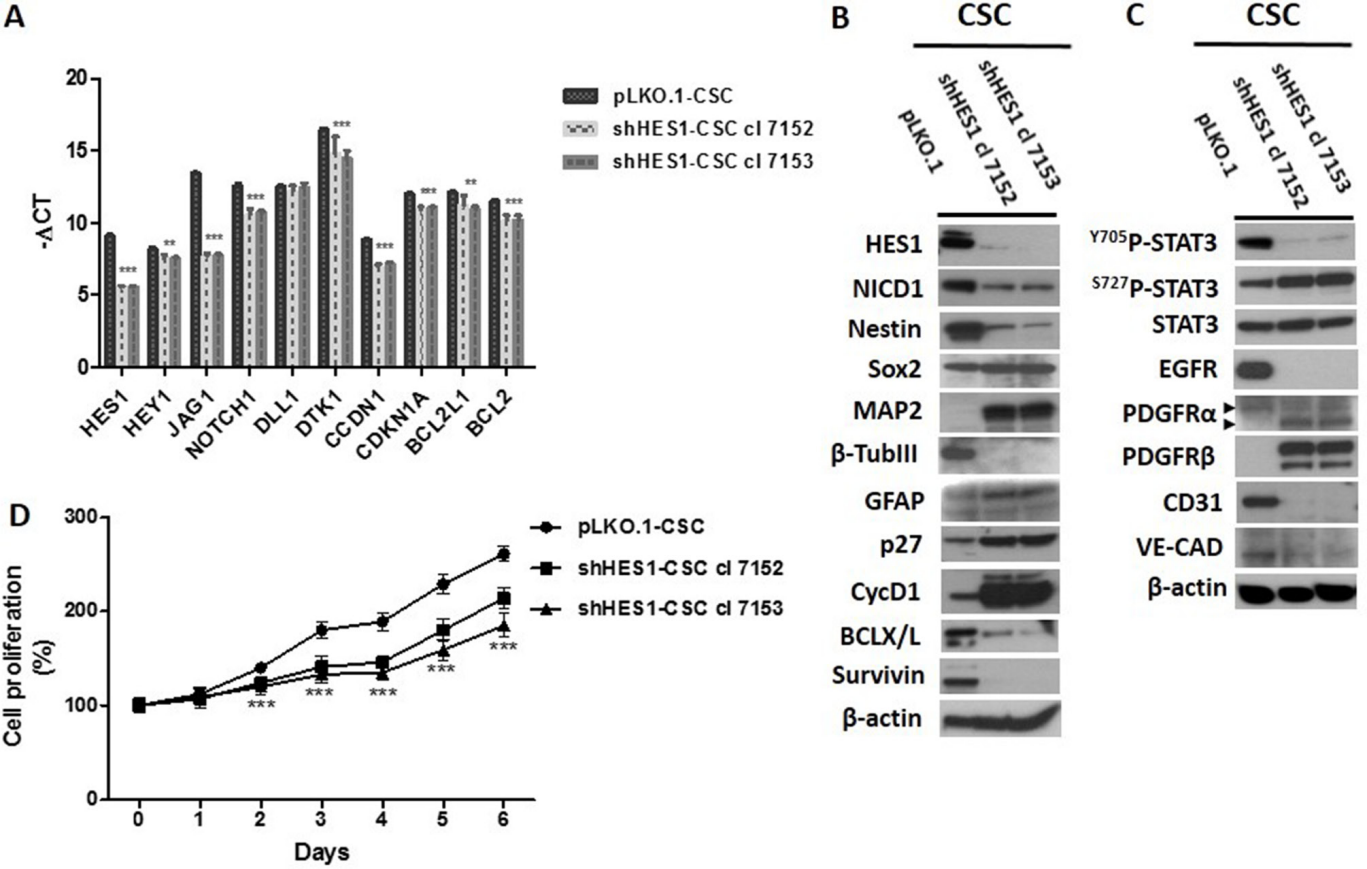
Downmodulation of Hes1 expression affects Notch1 signaling, self-renewal, oncogenic signaling pathways and cell growth rate in shHes1-CSC (**A**) RT-qPCR analyses reveal a significant decrease of Notch1 signaling components including conventional Hes1 targets. (**B**) Western blot analyses confirm the downmodulation of Notch1 signaling gene profile and highlight the neural differentiation of CSC via upregulation of MAP2 and GFAP and loss of Nestin. (**C**) Depletion of Hes1 diminishes the phosphorylation levels of Stat3 at Y705 but induces those at S727 residue. Furthermore, noteworthy are a remarkable reduction of EGFR protein the upregulation of PDGFRβ and the downmodulation of expression of angiogenic markers (CD31 and VE-cadherin). (**D**) Knockdown of Hes1 expression was associated with a highly significant inhibition of the proliferation rate of shHes1-CSC clone 7152 and 7153 *vs* pLKO.1 cells. Data are expressed as mean ± SD (*n* = 3), and are representative of three independent experiments. We denote the significant difference between cell clones and control cells (***P ≤ 0.001).

**Figure 2:**
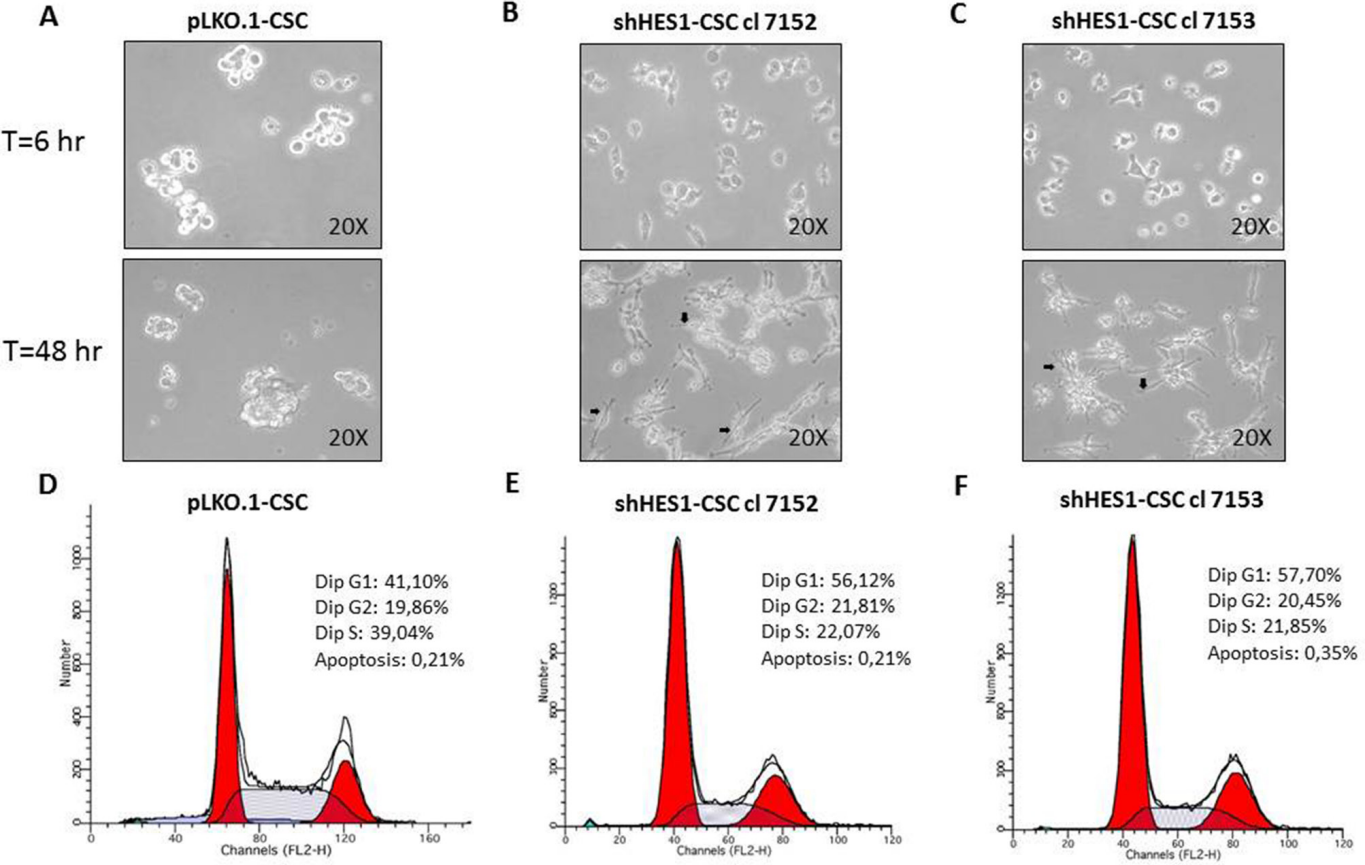
Targeting Hes1 expression induces morphological changes and negatively affects the cell cycle profile in shHes1-CSC (**A–C)** Phase-contrast images captured at 200× magnification after 6hs and 48hs in growth conditions, reveal substantial cell changes with attachment of shHes1-CSC on plastic dishes and formation of neuron-like cells (arrows in B,C), contrary to pLKO.1 cells which formed classical not-adherent neurospheres. (**D–F**) FACS analyses of cell cycle profiles reveal a substantial shift from S phase to G1 fraction of shHes1-CSC clones compared to pLKO.1 cells. Data of flow cytometry are representative of two independent experiments. The difference of percentages in cell cycle distribution between cell clones and p-LKO.1-CSC is consistent and can be considered biologically significant.

We highlighted the effects of Hes1-directed shRNA stably transduced in CSC by a lentiviral-based system (shHes1-CSC cl 7152 and 7153) *vs* control infected cells (pLKO.1-CSC) on key cellular pathways: Notch1 & RTKs signaling components, cell differentiation markers, cell cycle regulators, survival factors, and angiogenesis. Gene expression profile showed a significant down-modulation of several components of Notch1 signaling in shHes1-CSC in comparison to pLKO.1-CSC such as: Hairy and Enhancer of Split-1 (HES1), HES-Related Protein 1 (HEY1), Jagged1 (JAG1), NOTCH1, Deltex1 (DTK1), CyclinD1 (CCND1), Cyclin-Dependent Kinase Inhibitor 1 (CDKN1A), B-Cell Lymphoma-2 (BCL2) and BCL2-Like 1 (BCL2L1). The Delta Like Ligand 1 (DLL1) mRNA expression was similar between shHEs1-CSC clones and control cells (Figure 1A). Western blot assays confirmed the decrement of Hes1 and active Notch1 (NICD1) (Figure 1B). Unexpectedly, CycD1 protein was induced concurrently with p27, a cyclin-dependent kinase inhibitor that control the cell cycle progression at G0/G1. As a consequence of Hes1 depletion Survivin and Bcl-X/L protein levels were down-modulated (Figure 1B). As Notch1 is known to be a regulator for neurogenesis and plays crucial role in other cell fate decisions, our study clearly showed the upregulation of neuronal and glial markers MAP2 and GFAP respectively, and repression of β-TubIII and Nestin proteins in shHes1-CSC *vs* pLKO.1-CSC (Figure 1B). Accordingly to Huang et al., the activity of Notch1 is essential for Stat3 activation in mouse embryonic stem cells (mESC), and the authors suggest the presence of a dynamic equilibrium of Stat3 phosphorylation in Tyr705 (Y705) and Ser727 residues (S727) in the control of mESC fate. This prompted us to assess any change in Stat3 phosphorylation in shHes1-CSC (Figure 1B). shHES1-CSC clones displayed a weak phosphorylation at Y705 and an increase at S727, that correlated with the transition from the multipotent state to neuronal commitment of shHes1-CSC and manifested with low Nestin/high MAP2 expression respect to control cells (Figure 1B and Figure 2A–2C). Finally, we reported that Hes1-directed shRNA suppressed EGFR protein and upregulated PDGFRβ, but not PDGFRα (Figure 1B, 1C).

Notch pathway is known to be indispensable for vascular development during embryogenesis and postnatal angiogenesis and accordingly, we evaluated gene expression of endothelial markers such as CD31/CDH1 and the vascular endothelial cadherin (VE-CAD/CDH5) and we found that those were significantly affected by Hes1 depletion at the protein (Figure 1B) and mRNA level (Figure 4B).

**Figure 3:**
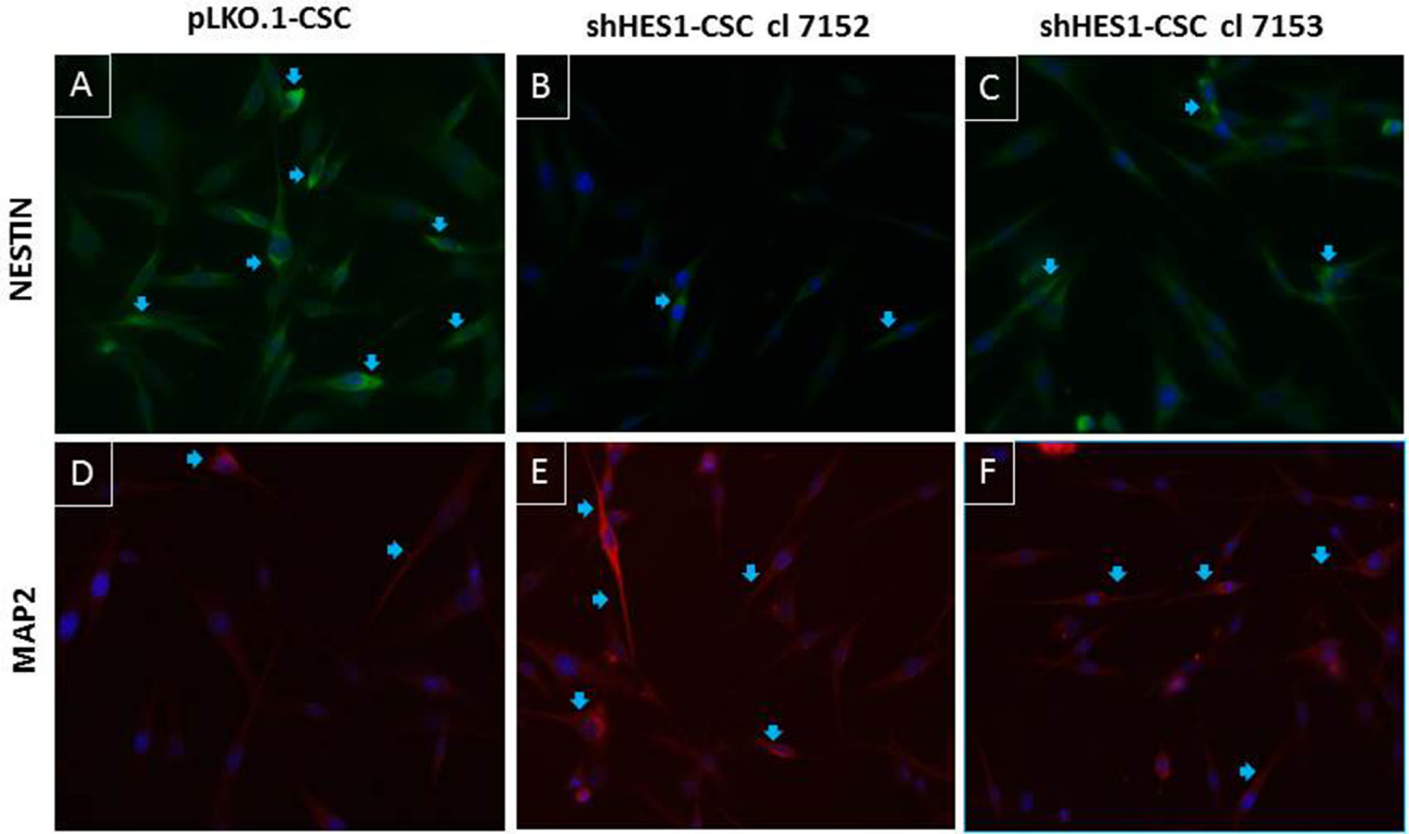
Modulation of neural differentiation in shHes1-CSC by immunofluorescence assay (**A–C**) Immunostaining for Nestin reveals its upregulation in control cells *vs* Hes1-depleted cells (arrows). (**D–F**) Immunostaining for MAP2 reveals its upregulation in Hes1-depleted cells *vs* control cells (arrows). Images were captured at 400X magnification.

**Figure 4:**
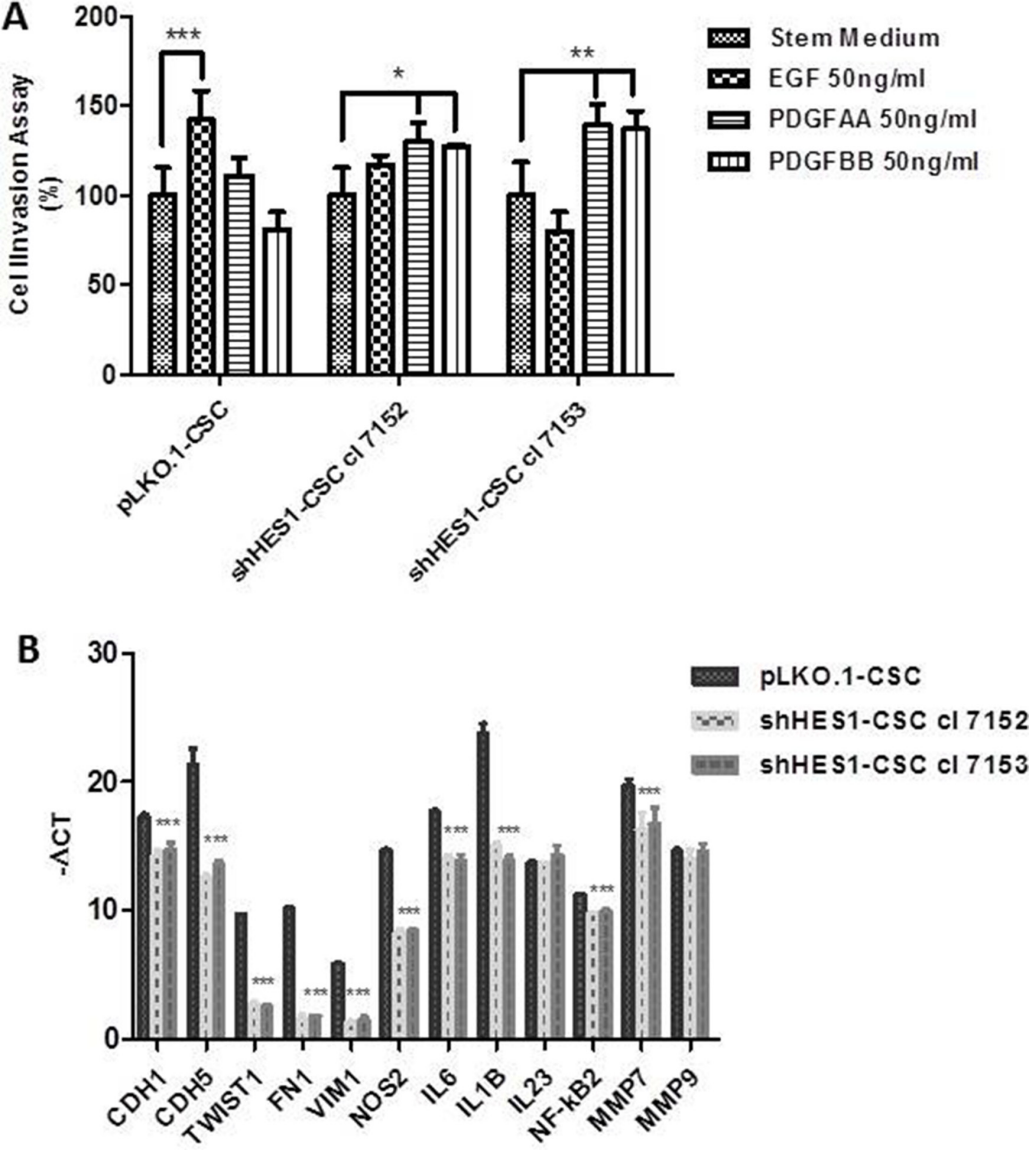
Targeting Hes1 impairs cell invasive abilities, modulates epithelial mesenchymal transition and inflammatory cytokines gene expression (**A**) Addition of EGF promoted a significant migration of pLKO.1 cells in cell invasion assay compared to stem medium, contrary to that occurs in shHes1-CSC. PDGFAA and PDGFBB promoted a significant migration of shHes1-CSC clones in comparison to stem medium, instead pLKO.1-CSC are not significantly influenced by PDGFAA and PDGFBB. Error bars represent the mean ± SD of two independent experiments performed in triplicate. *P values < 0.05, **P < 0.01**, ***P < 0.001 *vs*. control stem medium were considered statistically significant. (**B**) RT-qPCR analyses of key genes for angiogenesis, EMT, immunomodulation and cell invasion. mRNA expression for cell-substrate molecules interaction (CDH1, CDH5) were drastically downmodulated in shHes1-CSC *vs* pLKO.1 cells. Few EMT markers (TWIST1, FN1, VIM), together with immunomodulators (NOS2, IL6, IL1B, IL23, NF-κB2) and matrix metalloproteinases (MMP7, MMP9) were similarly downmodulated in shHes1-CSC in comparison to pLKO.1 cells.

### Targeting Hes1 expression decreases cell proliferation rate

To study the effects of Hes1 expression inhibition on cell proliferation we have used MTS assay. The clones 7152 and 7153 of shHes1-CSC showed a decrease of the cell proliferation rate vs pLKO.1-CSC, which was more pronounced in clone 7153 (Figure 1D). These findings indicated that there was a negative correlation between inhibition of Hes1 expression and GBM CSC proliferation, a finding that reinforce the importance of Notch signaling manipulation as a potential therapeutic target for GBM.

### Targeting Hes1 expression promotes neuronal commitment and modulates cell cycle profile

Following culture, pLKO.1-CSC formed classical non-adherent neurospheres (Figure 2A). In contrast, at 6 hs post culturing, the shHes1-CSC were shown to be adhered to the culture dish. At 48 hs post-culture, some of them exhibited a neuronal-like morphology with extended branched cytoplasmic processes (Figure 2B–2C). Immunofluorescence assays performed in cell proliferation conditions revealed that MAP2 (a/b) positivity in both clones of shHes1-CSC (Figure 3D–3F) inversely correlated with Nestin staining as compared to pLKO.1-CSC (Figure 1B and Figure3A–3C). A quantitative analysis of the cells immunostained for Nestin and MAP2 reported that p-LKO.1-CSC are 25% ± 4.2 and 5% ± 1.1 positives for Nestin and MAP2 respectively. In contrast, shHes1-CSC cl 7152 and cl 7153 are 9% ± 2.4 and 12% ± 3.1 % positives for Nestin respectively; instead 30% ± 3.2 and 25% ± 5.1 of positivity for MAP2 respectively. These findings might suggest a commitment of shHes1-CSC toward differentiation into neuronal lineage. In addition, we investigated the effects of Hes1 depletion on the modulation of the cell cycle profile (Figure 2D–2F). The G1, G2/M, S phases distribution for pLKO.1-CSC was 41.10%, 19.86%, 39.04% and apoptosis 0.21%, respectively (Figure 2D). For shHes1-CSC cl. 7152 was 56.12%, 21.81%, 22.07% and apoptosis 0.21% respectively (Figure 2E). For shHes1-CSC cl. 7153 it was 57.70%, 20.45%, 21.85% and 0.35% apoptosis, respectively (Figure 2F). These results showed that the knockdown of Hes1 expression induced a significant shifting of cells in G1 fraction, which correlated to the raising of p27 levels and the neuronal differentiation (Figure 1B and Figure 3D–3F).

### Depletion of Hes1 impairs cell invasive ability in response to epidermal growth factor

The important effects induced by Hes1 depletion on expression of fundamental transmembrane receptors may have implications in CSC invasion. This prompted us to evaluate, in transwell assay, the invasive properties of shHes1-CSC *vs* pLKO.1-CSC in response to EGF, PDGFAA and PDGFBB growth factors. We revealed a significant increase of invasion of pLKO.1-CSC in response to EGF. Conversely, silencing of Hes1 inhibited EGF-induced cell invasion and downregulation of MMP-7 and EGFR expression contributed to the reduced invasive ability of shHes1-CSC. MMP9 mRNA expression was identical among shHES1-CSC cell clones and pLKO. 1-CSC (Figure 4B). We further demonstrated that PDGF-AA and PDGF-BB dispensed alone improved shHes1-CSC migration compared to stem medium and pLKO.1-CSC (Figure 4A). Recent evidence have reported that Notch1 activation is associated with GBM proneural and mesenchymal subtypes, therefore by RTq-PCR analysis, we assessed whether well-known epithelial mesenchymal transition (EMT) genes were downregulated in shHes1-CSC. In fact, we highlighted a significant mRNA down-modulation of TWIST1, Fibronectin1 (FN1) and Vimentin1 (VIM1) in shHes1-CSC, indicating a role of Notch1 in defining the molecular profile of GBM mesenchymal subtype (Figure 4B).

A crosstalk between inflammatory mediators and Notch1 has been evidenced in colon cancer, glioma, cholangiocarcinoma and in macrophages activation. RTq-PCR analyses reported a significant down-modulation of inducible nitric oxide synthase (NOS2), pro-inflammatory cytokines (interleukin-6/IL6), interleukin-1β/IL1B). Conversely, IL23 mRNA expression was similar between shHes1-CSC and pLKO.1-CSC (Figure 4B).

### STAT3/5 is essential for survival of shHes1-CSC and pLKO.1-CSC

**Figure 5:**
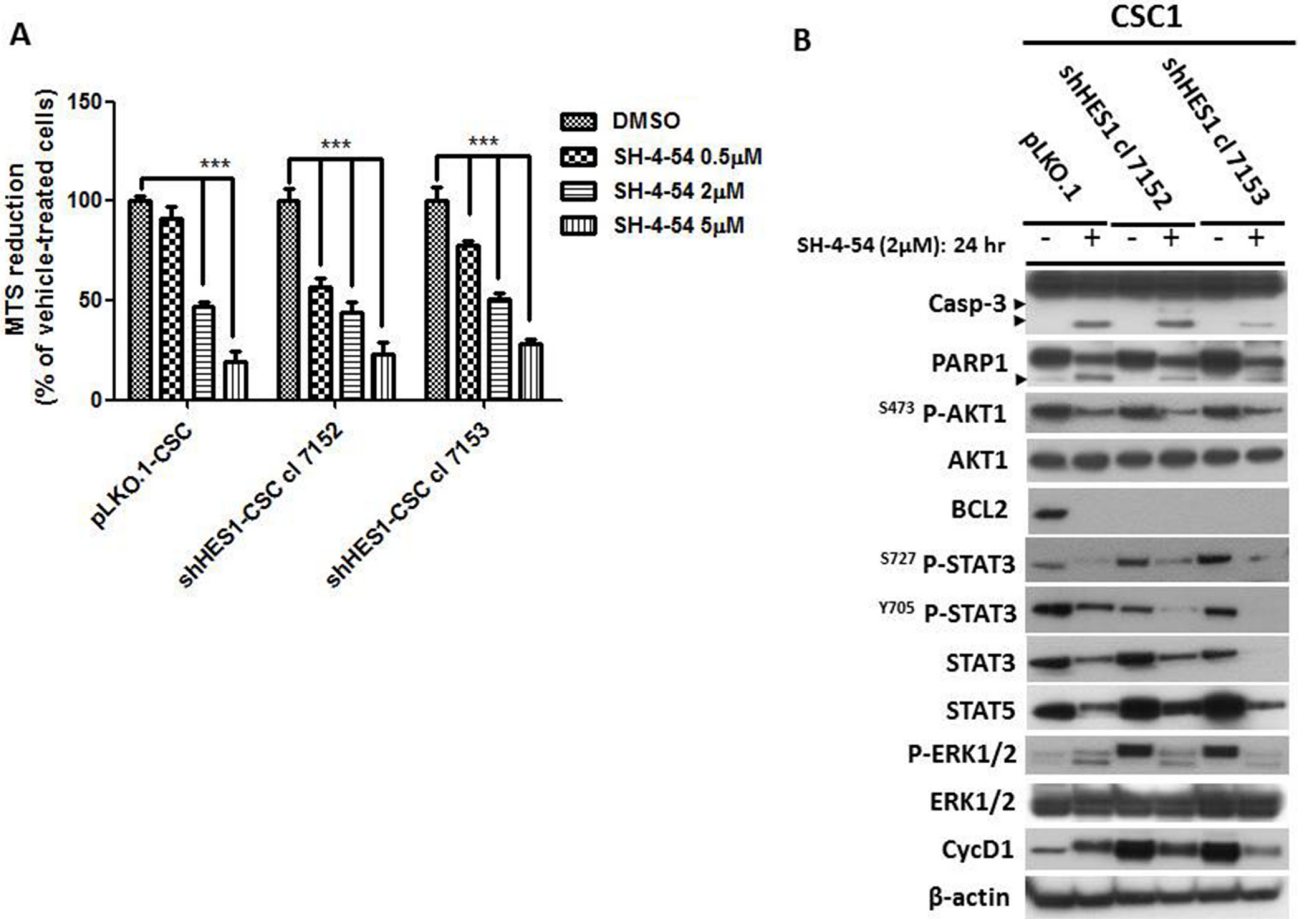
Stat3/5 are critical factors for survival of CSC (**A**) MTS assay was assessed to evaluate cells sensitivity to SH-4-54, a Stat3/5 inhibitor. CSC undergone three doses of SH-4-54 (0.5–2–5 μM) and tested for cell vitality after 24 h of treatment. Data are represented as mean ± SD (*n* = 3) and are representative of three independent experiments. ***P < 0.001 vs control was considered highly significant. (**B**) Western blot analyses reported the effects of SH-4-54 on Caspase-3 activation and PARP1 cleavage (arrowheads). Deleterious effects were observed for Akt, Stat3 and Stat5 and Erk1/2 signaling pathways, Bcl2 and CyclinD1.

The extensive detrimental effects produced by Hes1 depletion were not followed by CSC apoptosis. To reach this objective, we targeted Stat expression by using SH-4–54, a Stat3/5 small inhibitor. MTS assays were used to test the drug sensitivity of shHes1-CSC *vs* pLKO.1. shHes1-CSC were significantly sensitized at 0.5 μM of SH-4-54 compared to pLKO.1 after 1 day of treatment. Higher concentrations of SH-4-54 (2–5 μM) progressively prone CSC to cell death as seen by light microscope (data not shown) and MTS reduction output (Figure 5A). We used SH-4-54 at the intermediate dose, which reduced cell viability at fifty percent, to perform Western blots analysis. After 24 hs of SH-4-54 treatment, Stat3 and Stat5 proteins stability and activity were abolished (Figure 5B). SH-4-54 treatment was able to induce Caspase-3 activation (19-17 KDa) and PARP1 (116-89 KDa) fragments, and even anti-apoptotic signals such as P-Akt (S473) and P-Erk1/2 (T202-Y204), Bcl2 and CyclinD1 declined drastically (Figure 5B).

### Crenolanib is not effective in promoting apoptosis in shHes1-CSC

We previously reported that Crenolanib, a PDGFR inhibitor currently used in clinical trials to treat GBM, induced apoptosis at the same extent either in c-CSC1 or p-CSC1, circumventing the anti-Notch and anti-EGFR drug resistance. Herein, unexpectedly we reported that shHes1-CSC were not sensitized to Crenolanib as did pLKO.1-CSC (Supplementary Figure 1). Western blot data disclosed a significant upregulation of oncogenic and survival signals such as PDGFRβ, Stat5 and Erk1/2, and CyclinD1 in shHes1-CSC clones compared to pLKO.1-CSC (Supplementary Figure 1). Overall, our results revealed substantial differences in molecular pathways among the cell populations, and further studies are needed to better understand the signaling networks between these factors.

## DISCUSSION

Notch signaling has been demonstrated to have a central role in hematopoietic cancers and solid tumors, included GBM [24]. In our study, depletion of Hes1 inhibited proliferation rate of GBM CSC, which was more pronounced in shHes1-CSC cl. 7153 compared to pLKO.1-CSC. The reduction of cell proliferation most likely was due to cell cycle inhibitor p27 upregulation which drives shHes1-CSC clones to G1 fraction of cell cycle. Our finding indicate the importance of Notch signaling manipulation as a potential therapeutic target for GBM. The results presented here are partially in agreement with those of Saito et al., who demonstrated that Notch pathway inhibition by DAPT, a γ-secretase inhibitor, attenuated proliferation and self-renewal of glioma-initiating cells (GIC) and induced both neuronal and astrocytic differentiation [25]. In our work, the premise was how to explain the poor response of GBM CSC to GSI-X treatment with high Notch1 signaling expression. Conversely, Saito et al. reported that proneural GIC characterized by active Notch signaling components, i.e., Notch1, Notch3, Hes1, MAML1, Dll3, Jag2, and several other, exhibited great sensitivity to GSI treatment. Here, we strived to elucidate the mechanism(s) and the underpinning intracellular signaling pathways by which knockdown of Hes1 expression inhibits proliferation and attenuated invasive ability of GBM CSC. The knockdown of Hes1 expression was associated with downregulation of conventional Notch1 targets genes as BCL2, BCL2L1, CDKN1A, CCDN1 and few of the of them were also validated by Western blots. DTK1, a positive regulator of Notch1 signaling, was evenly significantly affected, although its mechanism of action has been shown independent of the signaling pathway involving RBP-Jκ and Hes1/Hes5. Deltex1 and Notch1 (NICD1) regulate negatively neuronal differentiation of neural progenitor cells by inhibition of the transcriptional activation of bHLH transcription factor MASH1 [26].

CyclinD1, implicated in G1 progression, was surprisingly upregulated in shHes1-CSC. CyclinD1 is a downstream target of multiple oncogenic pathways, e.g. Shh/Gli1 and Wnt/β-catenin [27, 28]. Intrinsically unstable, CyclinD1 is regulated by the ubiquitin-dependent proteasome system [29, 30]. Besides, Hes1 is known to directly suppress Gli1 transcription, therefore upregulating CyclinD1, suggesting that targeting Notch1 and Gli1 pathways simultaneously may be more effective to eliminate GBM cells [31].

Several evidence reported that *in vivo* hypoxia upregulated expression of Dll4, Jagged1, Notch1, Notch4, Hes1, and Hey which in turn promoted tumor angiogenesis [32, 33]. In addition, the upregulation of Dll4-Notch signaling components has been implicated in tumor angiogenesis but not in tumor growth [34]. Dll4 and Jagged1 may have opposing effects on tumor angiogenesis but a uniform prognostic effect in GBM [35]. Our results reported that the down-modulation of JAG1 and HEY1 gene expression in shHes1-CSC (except for DLL1) correlated to a decrease of angiogenic markers CD31 and VE-Cadherin.

In our study, a possible link between Notch/Hes1 signaling and the control of GBM growth could be through modulation of Jak2/Stat3 signaling pathway. In glioma stem-like cells, arsenic trioxide (ATO) inhibited the phosphorylation and activation of Akt and Stat3 through Notch signaling blockade. These data show that the ATO is a promising new approach to decrease GBM proliferation and recurrence by downregulation of Notch pathway [36]. The phosphorylation state of Stat3 plays a crucial role in self-renewal and differentiation of neural stem cell and embryonic stem cells (ESC) as well as in tumorigenesis [37]. It has been demonstrated that Stat3-Y705 is crucial for self-renewal of mESC, instead Stat3-S727 is decisive in the transition from pluripotency to neuronal commitment of mESC [38]. In addition, Hes1 physically interacts with Stat3 protein enabling Jak2 to phosphorylate Stat3 [39]. Consistent with these reports, we showed a positive correlation between Stat3 phosphorylation and Notch/Hes1 pathways. Accordingly, Hes1 depletion resulted in a reduction of Stat3 phosphorylation at Y705 and an increase at S727, which was accompanied by morphological modifications and appearance of MAP2 positive cells in hHes1-CSC *vs* control cells.

Constitutive activation of Stat3 and NF-κB signaling pathways regulate Notch pathway genes in GBM CSC [40]. Recent evidence reported that cAMP/PKA enhances IL-1β induced-IL-6 synthesis through Jak2/Stat3 activation, identifying novel therapeutic targets for the treatment of glioma [41]. Recent investigations report a crosstalk between inflammation and Notch1 signaling [42, 43] and highlight that inflammatory cytokines (IL1β, IL6, IL8 and p38 MAPK activity) are elevated in GBM [44]. In addition, in glioma-initiating cells and endothelial cells a new signaling PDGF-NOS2-ID4-miR129-Jagged has been discovered which drive to tumor progression [45]. Elevated NO synthase 2 (NOS2) expression correlates with decreased survival in human glioma patients [46]. Previously, we and others have demonstrated that NOS2 inhibition slowed colon and glioma stem cells growth *in vivo* animal models [47, 46]. Accordingly, we report that targeting Hes1 downregulates NOS2, IL1B, IL6 and NF-κB2 mRNA levels. NF-κB2 (p100/p52), a member of the NF-κB/Rel family of transcription factors, is implicated in a variety of genes important for immune function. Our results suggest a link between Notch1/Hes1 pathway and NF-kB2 gene regulation and corroborate Oswald’ results that reported that activated Notch1 induced NF-κB2 promoter activity via a functional RBP-Jκ binding site [15]. In addition, the non-canonical NF-κB signaling has been shown to contribute to tumorigenesis through reactivation of TERT gene [16].

Other research groups reported that knockdown of Notch1 expression by siRNA in U251 glioma cells downregulated the expression of EGFR, indicating the crosstalk and interaction of Notch1 and EGFR signaling pathways [48]. Consistent with the above report, we demonstrated a repression of EGFR protein in shHes1-CSC which justify their poor migration in response to EGF in cell invasion assay. Conversely, we noted an important upregulation of PDGFRβ in shHES1-CSC, but not for PDGFRα, that enable cell invasion in response to PDGF-BB and PDGF-AA respectively. This finding raises the issue that targeting Hes1 is not sufficient to impair CSC invasiveness features and suggest the need to correct the strategy of intervention. In fact, in our previous work, we stated that combination therapy either with anti-Notch/anti-EGFR or anti-EGFR/anti-PDGFR drugs compared to therapy alone was more effective in reducing survival of c-CSC compared to p-CSC, suggesting that in the area of GBM peritumor tissue occurs a selection process, which results a more aggressive cell phenotype [17, 18]. In fact, we earlier reported the upregulation, in p-CSC in comparison to c-CSC of several GBM samples, of drug resistance and/or EMT modulators such as TWIST, EDN1 and PRKCA [18]. In line with this, in the current study we reported a significant down-modulation of mesenchymal genes FN1, VIM1 and TWIST1 that positively correlated to the inactivation of Notch1 and Stat3 signaling pathways in shHes1-CSC, suggesting further the primary role played by the association of Stat3 and Notch1 in mesenchymal GBM [5–8].

MAP/Erk, a well-studied signaling pathway, is involved in basic cell processes such as cell growth, differentiation, migration and apoptosis. Recently Zhang et al., reported that valproic acid was able to induce apoptosis in U87 glioma cells through activation of Erk and Akt pathways and GSK3β inhibition [49]. Similarly, we earlier reported the up-regulation of multiple oncogenic signals in PDGFRα-depleted GBM CSC, i.e., CyclinD1, Erk1/2 phosphorylation, EDN1, PDGF-C, PDGF-D and PRKCB1 and so on. Simultaneously, we observed the start of neuronal differentiation, besides several detrimental effects on self-renewal, invasion, EMT and angiogenesis (Cenciarelli et al., 2016). In agreement with the just mentioned studies, we found that a marked activation of Erk1/2 in Hes1-depleted cells was linked to a significant reduction of proliferation rate and anti-apoptotic proteins expression (Bcl-2, Bcl-X), and finally the commitment toward neuronal differentiation. This behavior together with the upregulation of PDGFRβ and CyclinD1 might be a cell modality to survive and adapt to molecular changes induced by depletion of Hes1 and, therefore escape from apoptosis. Thus, further investigation of the crosstalk among PDGFR, CyclinD1 and Erk1/2 signal transduction pathways is essential for elucidating the mechanism(s) underlying the phenomena observed in shHes1-CSC.

The constitutive phosphorylation of Stat3 at Y705 seen in pLKO.1-CSC and its reduction following Hes1 depletion, prompted us to assess the effects of a pharmacological blockade of Stat3/5 on survival of GBM CSC. We succeeded to promote apoptosis in both cell populations through loss of Stat3 and Stat5 proteins, besides a marked decline of p-Akt1 and p-Erk1/2 phosphorylation. Since shHes1-CSC displayed a less aggressive behavior respect to control cells, we assessed whether those cells would be more sensitive to Crenolanib (CR), a PDGFR inhibitor, respect to pLKO.1-CSC. CR was able to induce apoptosis in control cells as earlier reported (Cenciarelli et al., 2014), but not in shHes1-CSC. This finding could be explained by the lack of effectiveness by CR on the constitutive oncogenic signals, which included PDGFRβ, Stat5, Erk1/2 and CyclinD1. Future investigations should pay attention to the multiple effects generated by anti-cancer drugs, and although those are developed for specific molecular targets, cells are able to find new modality to escape the drug's effect.

## MATERIAL AND METHODS

### Ethical statement

Procedures for collection of adult human GBM CSC were approved by the Ethical Committee of the Catholic University of Rome as reported previously [23]. Informed consent was obtained and all patients were fully aware of the aims and scope of this work. The ethical principles of the declaration of Helsinki were strictly followed.

### Cell culture and drug treatment of glioblastoma stem cells

We have used the same clinical materials reported in our previous papers [17, 23]. In brief, the CSC cells were retrieved from adult patients affected by GBM and undergoing craniotomy at the Institute of Neurosurgery, Catholic University-School of Medicine of Rome, Italy. Dissociated cells were cultured in the presence of human recombinant EGF (20 ng/ml; PeproTech, Rocky Hill, NJ), human recombinant bFGF (10 ng/ml; PeproTech), in DMEM/F12 (1:1) serum-free medium (Invitrogen, Carlsband, CA) as reported previously (Cenciarelli et al. 2014). Floating and adherent neurospheres were dissociated with Accutase at 37°C (Merck-Millipore). For the drug treatments, 5 μM of Crenolanib (CP-8685596) and SH-4-54 (Selleckchem) were added for 1 day.

### shRNA, transfection and lentivirus production

The experiments of Hes1 RNA interference were performed using Mission Lentivirus-based shRNA (NM_005524.2-Sigma-Adrich). Lentivirus production was performed as reported previously [17]. We selected several puromycin resistant Hes1-directed shRNA cell clones. shHES1-CSC clone 7152 and 7153 were selected based on significant down-modulation of Hes1 expression.

### Western blots

GBM CSC are seeded as single cells (1 × 10^6^/p90 dish) in proliferation medium and collected 2–3 days later for Western blot analysis. Cells pellet were lysed for 30 min in 200 μl of lysis buffer (1% NP-40, 0.01% SDS, 20 mM Tris–HCl pH 7.4, 300 mM NaCl, 1 mM EDTA, 1 mM Na_3_VO_4_ and protease inhibitors cocktail from Sigma–Aldrich). Cells are ultrasonicated with two pulses of 5 sec at 50% of amplitude (Sonics and Materials, Newtown, CT). Equal amounts (30 μg/lane) of total protein extracts, quantified by Bio-Rad protein Assay (Bio-Rad, Munchen, Germany), were loaded on Bolt 4-12% Bis-Tris gels (Invitrogen), and transferred on Hybond-P Extra membrane (Amersham Biosciences, GE Healthcare). Filters were immunoblotted using the following primary antibodies: goat anti-PDGFRα and mouse anti-β-actin (SIGMA), rabbit anti-EGFR, anti-PDGFRβ, anti-T202/Y204-ERK1/2, anti-ERK1/2, anti-Y705-STAT3, anti-S727-STAT3, anti-STAT3, anti-STAT5, and anti-Caspase-3 (all purchased from Cell Signaling, USA), mouse anti-S473-AKT1 and rabbit anti-AKT1 (Calbiochem), mouse anti-BCL2 (Dako), mouse anti-Nestin (Millipore), mouse anti-GFAP (Covance), and mouse anti-βTubIII (Chemicon), mouse anti-MAP2 (a/b) (Millipore), mouse anti-BCL2 (DAKO), goat anti-VE-CAD, rabbit anti-CD31, anti-BCL-X/L, anti-CycD1, anti-p27, anti-SOX2 and anti-PARP1 (all purchased from Santa Cruz-USA), rabbit anti-Survivin (Abcam). After three washing with TBS-Tween buffer, immuno-reactive proteins were detected using rabbit anti-mouse, donkey-anti-rabbit and donkey anti-goat horseradish peroxidase-conjugated secondary antibodies directed to the appropriate primary antibodies (Jackson Immunoresearch Laboratories, West Grove, PA). The proteins were then visualized using the chemiluminescence system (Millipore). Gels and Images acquisition was done by HP Photosmart Essential Ver. 1.12 and Adobe Photoshop CS5 respectively.

### Cell proliferation and cytotoxicity assays

We used the CellTiter 96 Aqueous One Solution Reagent (Promega), a cell proliferation colorimetric assay containing a novel tetrazolium compound MTS. shHES1-CSC cells *vs* control cells (pLKO.1) are dissociated into single cells and 2 × 10^4^ cells/well plated in triplicate on 6-well dishes. Cells were harvested and counted at different time points in growth medium, considering the starting time the day after plating. For pharmacological studies, 5 × 10^4^ cells/well were plated in triplicate for each group on 12-well plates and treated for 1 day with the drugs or DMSO-vehicle. The metabolically active cells reduced MTS into a soluble formazan product, whose absorbance is measured at 490 nm in a plate reader (Bio-Rad). These experiments were performed twice and each time in triplicate. The absorbance values of the collected samples were subtracted from the background absorbance of medium-only control and expressed as mean average ± SD (*n* = 3).

### Immunocytochemistry

Cell populations were dissociated with Pasteur pipettes and plated onto Matrigel GFR-coated glass coverslips (BD-Italia) to perform the immunostaining. Cells were fixed in 4% paraformaldehyde and permeablized with 0.2% Triton-100 and subsequently processed for immunolabeling. The following primary antibodies (Ab) were used: mouse anti-MAP2 (a/b), and rabbit anti-Nestin (Millipore). The secondary Ab used were: fluorescein isothiocyante (FITC) affinity purified goat anti-rabbit and Tetramethyl rhodamine isothiocyanate (TRITC) affinity purified donkey anti-mouse (Chemicon). Nuclei were stained with Hoechst 33258 diluted in PBS (0,2 μg/ml; SIGMA). Cells were photographed at 400X magnification using a fluorescent microscope (Olympus microscope OLYMPUS Bx5 with Spot CCD Camera). A quantitative cell analysis was performed by counting for each antibody used almost 100 cells/field of five fields from each of three independent experiments.

### RT-qPCR assay

Total RNA was extracted using Triazol and by RNeasy mini kit (Qiagen, USA). cDNAs were obtained using QuantiTect Reverse Transcription kit (Qiagen, USA). Quantitative Real Time Reverse Transcriptase PCR (RT-qPCR) was conducted using SYBR Hi-ROX kit (Bioline, UK). RT-qPCR was performed by 7900HT instrument equipped with SDS2.2 software (Applied Biosystems, CA). The sequences of oligonucleotides used for RT-qPCR were described in Table 1. Relative levels of gene expression (-ΔCt), were normalized respect to housekeeping genes (GAPDH, TBP) by using the ΔCt method following manufacturer's guide.

**Table 1:** List of primers for RT-PCR analysis

Gene ID	Name		Sequences 5′ 3′
NM_005524.3	hHES1	For	AAGAAAGATAGCTCGCGGCA
		Rev	CGGAGGTGCTTCACTGTCAT
NM_001040708.1	hHEY1	For	AGGCTGGTACCCAGTGCTT
		Rev	GCATTCCCGAAATCCCAAACT
NM_000214.2	hJAG1	For	ATGGGCCCCGAATGTAACAG
		Rev	ATCACAGTACAGGCCTTGCC
NM_017617.4	hNOTCH1	For	CACTGCGAGGTCAACACAGA
		Rev	GTCCACATCGTACTGGCACA
NM_005618.3	hDLL1	For	GGAGAAAGTGTGCAACCCTG
		Rev	CCCACTCTGCACTTGCATTC
NM_001317172.1	hDLK1	For	TTCACCGCAAGAGGATTCCC
		Rev	GTTGGACGTGCCGATAGTGA
NM_053056.2	hCCDN1	For	CAGATCATCCGCAAACACGC
		Rev	ATGGAGGGCGGATTGGAAAT
NM_000389.4	hCDKN1A	For	TGCCGAAGTCAGTTCCTT
		Rev	CATTAGCGCATCACAGTC
NM_000633.2	hBCL2	For	CTTTGAGTTCGGTGGGGTCA
		Rev	GGGCCGTACAGTTCCACAAA
NM_001191.3	hBCL2L1	For	AGCTTTGAACAGGATACTTTTGTGG
		Rev	GGTGGGAGGGTAGAGTGGAT
NM_001077494.3	hNF-κB2	For	GGCCGGGACAAGAGAAAAGA
		Rev	CCAGAATTTTAGACGCCCGC
NM_000625.4	hNOS2	For	TGAACTACGTCCTGTCCCCT
		Rev	CTCTTCTCTTGGGTCTCCGC
NM_000600.4	hIL6	For	TTCGGTACATCCTCGACGGC
		Rev	CAGTGCCTCTTTGCTGCTTTC
NM_000576.2	hIL1B	For	AGCTGATGGCCCTAAACAGAT
		Rev	TGGTCGGAGATTCGTAGCTG
NM_016584.2	hIL23A	For	CCGCTTCAAAATCCTTCGCA
		Rev	CTGCCTTTAGGGACTCAGGG
NM_002423.4	hMMP7	For	AGAGATCCCCCTGCATTTCA
		Rev	GGCCCATCAAATGGGTAGGAG
NM_004994.2	hMMP9	For	CGACGTCTTCCAGTACCGAG
		Rev	TTGTATCCGGCAAACTGGCT
NM_003380.3	hVIM	For	AGAGGAAGCCGAAAACACCC
		Rev	TCAAGGTCAAGACGTGCCAG
NM_000474.3	hTWIST	For	TCAAGAGGTCGTGCCAATCA
		Rev	ATGGTTTTGCAGGCCAGTTT
NM_001306132.1	hFN1	For	TGACAAGCAGACCAGCTCAG
		Rev	CTGTCACACGAGCCCTTCTT
NM_001795.3	hCDH5	For	ATGCGGCTAGGCATAGCATT
		Rev	TGTGACTCGGAAGAACTGGC
NM_004360.3	hCDH1	For	CGAGAGCTACACGTTCACGG
		Rev	CTTTGTCGACCGGTGCAATC
NM_001172085.1	hTBP	For	GAACATCATGGATCAGAACAACA
		Rev	ATAGGGATTCCGGGAGTCAT
NM_001256799.2	hGAPDH	For	AGCCACATCGCTCAGACA
		Rev	GCCCAATACGACCAAATCC

### Cell invasion assay

For the invasion assay, 1 × 10^5^ cells were loaded in 0.3 ml of Stem Medium in triplicate into the top chamber of 150 μl matrigel-coated transwell insert (Millipore). The bottom wells contained 0.4 ml of stem medium as control or medium plus chemoattractant (EGF or PDGF-AA or PDGF-BB). After three days, cells on the top surface of the filter are removed with a cottonswab. Thereafter, the filters were fixed and stained with crystal violet and subsequently washed to collect the staining solution. OD values, proportional to the number of cells, were measured on a plate reader by a 590 nm filter (Bio-Rad). These experiments were performed twice and each time in triplicate. The absorbance values were calculated as mean ± SD (*n* = 3).

### FACS analysis

Cells were fixed with 0.5 ml of cold Methanol/Acetone Solution (1:5) and left at 4°C for at least 1 hour, then centrifuged at 950 RPM for 5 minutes. The cell pellet was resuspended in 100 μl of PBS, 375ul of RNAse A (100 μg/ml) and 25 μl of Propidium Iodide (1 mg/ml). Cells were incubated at room temperature for 15 minutes and left in the dark at 4°C until FACS analysis. Samples were acquired on FacsCalibur (BD Becton Dickinson) at 488 nm. The acquired FACS data were analyzed by ModFit LT software (Verity Software House, Inc.) to determine the percentage of cells sub G1, G1, S and G2 /M phases. These experiments were performed twice for each sample.

### Statistical analysis

Statistical analysis was performed with Prism5 (GraphPad) and Microsoft Office Excel 2013. All data shown are representative of results obtained from experiments conducted two or three times as specified in the specific sections. The results were analyzed by Two-way ANOVA and Bonferroni's posttests. Data are expressed as mean ± standard deviation (SD) and *P* values ≤ 0.05 (*), ≤ 0.01(**), ≤ 0.001 (***) were considered statistically significant.

## CONCLUSIONS

We used shRNA to knockdown Hes1 expression in GBM CSC to understand its mechanism(s) of action and make available further therapeutic targets against GBM. Our findings provide the rationale for possible targeting of Notch pathway through inhibition of Hes1, which would be beneficial in modulation of the cell growth and invasive ability of GBM CSC, but not sufficient to induce CSC apoptosis. To conclude, we suggest that a Stat targeted therapy, would provide more therapeutic effects than an anti-Notch/Hes1 drug intervention.

## SUPPLEMENTARY MATERIALS FIGURES


