# Supplementary material for: The interference of Notch1 target Hes1 affects cell growth, differentiation and invasiveness of glioblastoma stem cells through modulation of multiple oncogenic targets
Source: Oncotarget. 2017 Feb 2;8(11):17873–86. doi: 10.18632/oncotarget.15013 (PMC5392293; doi:10.18632/oncotarget.15013)
Supplement: Supplementary file 1 [file oncotarget-08-17873-s001.pdf]

The Interference of Notch1 target Hes1 affects cell growth, differentiation and invasiveness of glioblastoma stem cells through modulation of multiple oncogenic targets

Supplementary Materials

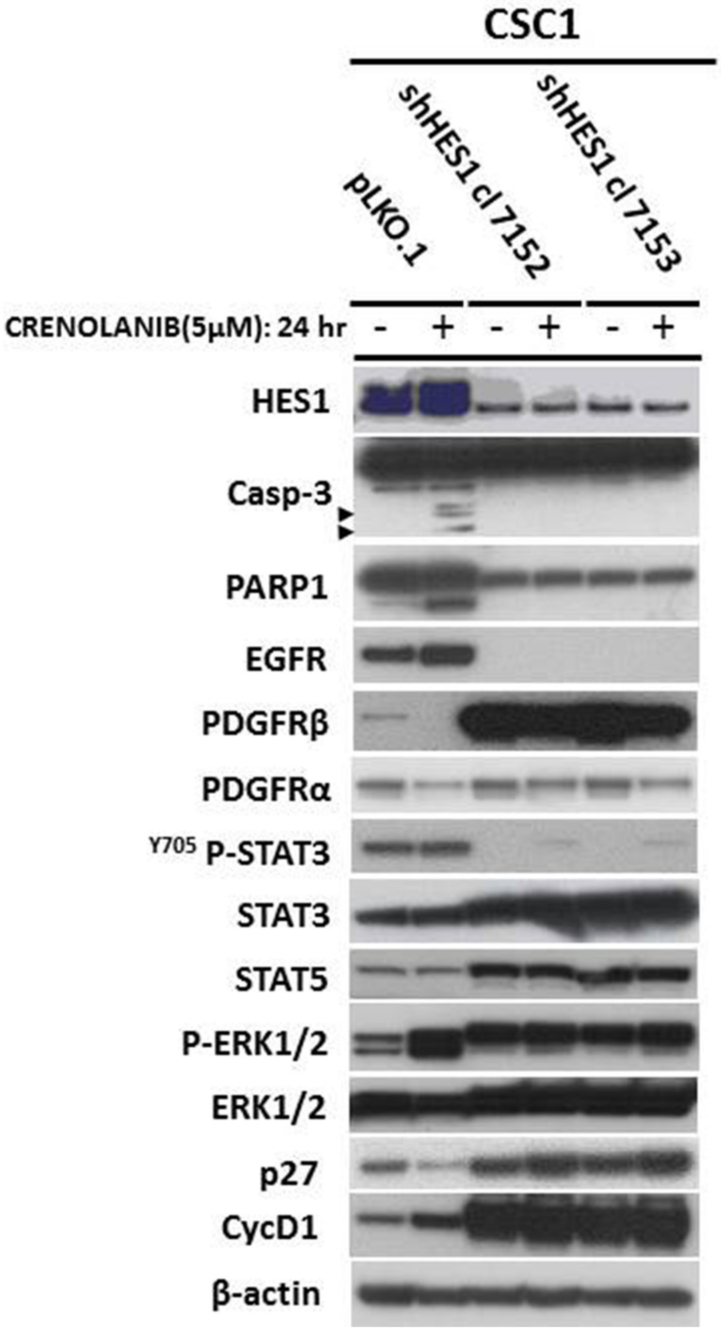

Supplementary Figure 1: Inhibition of PDGFR signaling is not crucial to induce apoptosis in shHes1-CSC. Crenolanib (CR), a PDGFR inhibitor, promotes apoptosis in pLKO.1 cells by modulation of PDGF receptor α/β; p-Erk1/2 and CyclinD1 were upregulated by CR as earlier reported. Conversely, CR does not produce apoptosis in Hes1-depleted cells. The protein levels of the major oncogenic signals (PDGFRα/β, STAT5, Erk1/2, CyclinD1) were not changed in shHes1-CS after 24 h of CR treatment compared to not treated cell.
